# Changes in cannabis use and associated correlates during France’s first COVID-19 lockdown in daily cannabis users: results from a large community-based online survey

**DOI:** 10.1186/s12954-022-00611-x

**Published:** 2022-03-15

**Authors:** Salim Mezaache, Cécile Donadille, Victor Martin, Maëla Le Brun Gadelius, Laurent Appel, Bruno Spire, Laelia Briand Madrid, Martin Bastien, Perrine Roux

**Affiliations:** 1 Sciences économiques & sociales de la santé & traitement de l’information médicale, Aix-Marseille Univ, INSERM, IRD, SESSTIM, Marseille, France; 2 Association Bus 31/32, Marseille, France; 3 Faculté de médecine, INSERM U1252 SESSTIM, 27 bd Jean Moulin, Marseille, France

**Keywords:** Cannabis, COVID-19, Quarantine, Mental health, Pain, Sleep disorders

## Abstract

**Background:**

Lockdown measures during the first wave of the COVID-19 pandemic in France led to serious public health concerns over people who use illicit drugs, especially in terms of mental health. We assessed changes in cannabis use during the first lockdown in France among daily cannabis users and associated correlates.

**Methods:**

CANNAVID is a French, national, cross-sectional web-based survey, conducted from 17 April to 11 May 2020. Daily cannabis users aged ≥ 18 years and living in France were invited to participate through advertisements. Respondents completed an ad hoc questionnaire on a dedicated online platform. We analyzed changes in cannabis use during the first lockdown (*i.e.,* stopped, decreased, unchanged, or increased) and performed a multinomial logistic regression analysis to evaluate correlates of these changes.

**Results:**

Of the 4019 participants, 74.0% were men. Median age was 27 years (interquartile range: 22–37). With regard to cannabis use, 293 (7.3%) persons stopped, 1153 (28.7%) decreased, 1146 (28.5%) did not change, and 1427 (35.5%) increased their use during the lockdown. A multinomial logistic regression model revealed several sociodemographic, behavioral and health-related factors associated with changes in cannabis use. Compared with participants with an unchanged level of cannabis use during the lockdown, those who increased and those who stopped cannabis use were more likely to have increased tobacco and alcohol use and to have experienced depression and sleep disorders intensification. Those who stopped cannabis use were also more likely to have increased benzodiazepine use and to have experienced pain increase during lockdown.

**Conclusions:**

France’s first COVID-19-related lockdown had a differential impact on daily cannabis users’ consumption patterns. Most study respondents reported changes to their cannabis consumption pattern. Those who reported a stable cannabis use were more likely to report fewer negative changes. Specific interventions are needed for this population, as well as research to assess the long-term impacts of these changes.

## Introduction

The health emergency caused by the coronavirus disease 2019 (COVID-19) pandemic prompted the French government to implement lockdown measures to control its spread [1]. The country’s first lockdown ran from 17 March to 11 May 2020. Measures included the closure of schools and non-essential retail shops, as well as stay-at-home orders and travel restrictions. People were only allowed to leave their homes for work (through exemptions), essential shopping, health issues, urgent family needs and physical activity. The latter was restricted to one hour per day within a 1 km radius from home [2]. Failure to comply with these rules was sanctioned by a 135€ fine [3]. This lockdown rapidly affected the everyday lives of the French population at the socio-professional, economical, behavioral and psychological levels [4, 5]. Concerns for people in France who use illicit drugs arose following preliminary results from qualitative surveys, drug addiction monitoring system reports and community alerts [6, 7], which highlighted substantial changes in the illicit drug market, specifically the closure of dealers’ points of sale, supply shortages and a surge in prices. These findings reflected those for certain areas in the United States of America, where an immediate decrease in cannabis and methamphetamine seizures was observed during lockdown because of less availability [8]. Worldwide, changes in drug use patterns—both increases and decreases—were reported for different general populations [9–11].

Despite France’s repressive policy on cannabis, it is the most widely consumed illicit drug in the country. In 2017, a national representative survey estimated that almost half of the French population aged between 18 and 64 years old reported lifetime cannabis use while one in ten reported consuming the drug in the previous month [12]. It was also estimated that there are approximately 900,000 daily cannabis users (CU) in France. While a large proportion of CU are recreational users [13], some (also) use the drug for therapeutic purposes. Indeed, despite its association with physical and cognitive risks [14, 15], the therapeutic benefits of cannabis for chronic pain [16], spasticity in multiple sclerosis [17] and chemotherapy-induced nausea and vomiting [18] are widely recognized. These benefits underline why several studies have reported that CU use the drug to relieve physical and psychological symptoms associated with chronic diseases [19], pain [20], insomnia [21], anxiety and depression [22], and loss of appetite [23].

In the context of the ongoing COVID-19 pandemic (as of September 2021), many uncertainties exist over the impact of the pandemic and associated restrictions on the overall health situation of people who use drugs. Understanding the changes in cannabis users’ patterns of use and health outcomes during France’s first lockdown could be very beneficial to understand current behaviors and adapt prevention messages. While some studies conducted during the first lockdown in other countries provided data for regular CU, they either focused on therapeutic use [24, 25], or were conducted in a context where national drug policy differs from that in France [26]. The purpose of our study was to investigate the impact of the first COVID-19 lockdown on daily cannabis users—whether recreational or therapeutic—in France. More specifically, we aimed to describe changes in their cannabis use, and to assess correlates associated with these changes.

## Methods

### Study design and participants

CANNAVID is a French, national, cross-sectional, web-based survey conducted between 17 April 2020 (one month after the country’s first lockdown started) and 11 May 2020 (the last day of the first lockdown). This community-based participatory research study was designed and implemented in collaboration with the harm reduction center ‘Bus 31/32’ and with cannabis users’ community associations. In order to solicit participation, the study was advertised through social media, cannabis community websites and mainstream press and radio. Eligibility criteria were being aged ≥ 18 years, living in France, and using cannabis daily. All participants received information before enrolment. Ethical approval was granted by the French national institute of health, and by the medical research ethics committee in Paris (IRB 00003888, N°20-676). The study did not receive external funding. No personal data which could have led to the identification of study participants (*e.g.,* names, IP addresses) were collected.

### Data collection and outcomes

Data were collected in a 15-min, self-administered questionnaire on a dedicated online platform (LimeSurvey.org). They were grouped into three different categories as follows: (i) *sociodemographics*: age, gender, education level (< secondary school diploma *vs.* ≥ secondary school diploma), living with a partner during the lockdown (‘yes’/‘no’), living with children during the lockdown (‘yes’/‘no’), number of people living in the house (including the participant) before and during the lockdown, having an external space in one’s housing (*e.g.,* large terrace, garden) (‘yes’/‘no’) before and during lockdown, town/city of residence before and during lockdown; (ii)* cannabis use (before and during the lockdown)*: median number of daily intakes, form used (dried flowers *vs.* resin *vs.* other (oil, e-liquid, etc.)), route of administration (smoking (joint with/without tobacco) *vs.* other (ingestion, vaporization)), supply route (home-grown cannabis *vs.* other), stocking up on cannabis before the lockdown (‘yes’/’no’), therapeutic use of cannabis before the lockdown (three categories: (1) ‘not always’,(2) ‘always’ and (3) ‘doesn’t known’ or ‘missing’); (iii) *substance use other than cannabis (specifically,* t*obacco, alcohol and benzodiazepines):* number of intakes per day and number of days per month (before and during the lockdown), alcohol consumption using the Alcohol Use Disorders Identification Test (AUDIT-C) questionnaire [27] (during the lockdown); (iv)* health status*: self-reported chronic or acute mental illness before the lockdown (either psychological illness or addiction-related illness) (‘yes’/’no’), chronic or acute physical illness before the lockdown (‘yes’/’no’), experiencing symptoms suggestive of COVID-19 since the beginning of the pandemic (*i.e.,* fever, cough, sore throat, anosmia, ageusia, headache and dyspnea).

Using all these items we built the following variables:

*Changes in cannabis use (principal outcome):* To assess changes in cannabis use before and during the lockdown period, we compared the number of daily intakes between the two timeframes. A four-category variable was created as follows: (i) stopped, (ii) decreased, (iii) unchanged, and (iv) increased. This constituted the main exposure variable for the study. Participants were classed in the ‘stopped’ category if they completely stopped cannabis use during the first lockdown. The ‘decreased’ category included people who no longer used cannabis daily and those who reported daily cannabis use but fewer daily intakes than before the lockdown. Participants who reported the same daily cannabis use as before the lockdown were classed in the ‘unchanged’ category. Finally, the ‘increased’ category comprised participants who reported more daily intakes during the lockdown than before it. It is important to point out that we measured the number of daily intakes not the quantity of cannabis used daily.

*Changes in the use of psychoactive substances other than cannabis:* For each substance, we combined the number of intakes per day and the number of days per month and compared the monthly frequency (i.e., number of daily intakes by number of days) before and during lockdown (‘increase’ *vs.* ‘decrease or unchanged’).

*Type of area of residence* Using the French National Institute of Statistics and Economic Studies (INSEE) notion of the ‘urban unit’ [28], the type of town/city of residence was categorized into an urban (urban unit > 200 k inhabitants), semi-urban (urban unit between 10 and 200 k inhabitants) or rural (urban unit < 10 k inhabitants or rural town) area, according to INSEE’s data for 2020.

*Change in employment status:* We combined employment status before and during the first lockdown to build a four-category employment variable as follows: (1) teleworking (since lockdown), (2) partial unemployment/receiving a payroll subsidy/job loss/sick leave/disability, (3) unchanged employment status (whether employed or not) (reference category) and (4) student (before the lockdown only).

*Symptoms suggestive of COVID-19*: (1) ‘no symptoms’ (reference)*,* (2) ‘mild symptoms’, (3) ‘dyspnoea’.

*Health outcomes during lockdown*: These included anxiety, depression, sleep disorder intensification and pain increase. Anxiety and depression were only measured for during the lockdown, not before it. We used the Hospital Anxiety and Depression (HAD) scale, with the commonly used cut-off score ≥ 8 (‘yes’) to identify clinically-significant disorder for both conditions [29]. Participants who reported experiencing more sleep disorders during the lockdown than before it were classified as having sleep disorder intensification (‘yes’) *versus* all others (the same, fewer, no such disorders) (‘no’). Similarly, pain increase was defined as an increase in pain during the lockdown with respect to before it (‘yes’/‘no’).

### Statistical analysis

The first step of the analysis was to describe the characteristics of participants with respect to cannabis use changes using descriptive statistics according to several socio-demographic (during the lockdown), behavioral and health factors. We used a multinomial regression model to assess correlates of a change in cannabis use patterns using the four-category variable described above (i.e., the principal outcome). The ‘unchanged’ category was the reference comparison category for the other three categories (i.e., stopped, decreased, and increased). After performing a univariable analysis, we preselected variables which had at least one category associated with the outcome, defined by a *p* value < 0.10. We then built a multivariable model using a backward selection procedure to identify the best model by removing variables one at a time with a *p* value of > 0.05. Missing data for all covariates were treated as a separate category in the descriptive table and in the regression models.

Overall, 4279 participants completed questionnaires on the online platform. Of these, 134 were excluded because their town/city of residence during the first lockdown was located outside France. A further 126 were excluded because of missing data for cannabis use frequency before or during the lockdown. The study sample therefore comprised 4019 participants.

## Results

### Study sample

Respondents’ characteristics are described in Table 1. Men accounted for 74.0% of the sample and median age was 27 years (interquartile range (IQR): 22–37; min–max: 18–74 years). Most respondents had at least a secondary school diploma (82.0%) and were living in an urban area during the first lockdown (55.9%). With regard to health, 13.0% had one or more reported chronic or acute pre-existing physical illnesses, while 9.6% had pre-existing chronic or acute mental health disorders. One in five reported prevalent chronic pain. Cannabis was mainly smoked (95.7%) and dried flowers were the most common form of administration (62.6%). Over one in ten participants reported always using cannabis for therapeutic purposes.

**Table 1:** Characteristics of respondents according to changes in cannabis use patterns during the first COVID-19-related lockdown in France

	All (*n* = 4019)	Change in cannabis use during lockdown				p
		Stopped (*n* = 293)	Reduced (*n* = 1153)	Unchanged (*n* = 1146)	Increased (*n* = 1427)	
	*n* (%) or median [IQR]	*n* (%) or median [IQR]	*n* (%) or median [IQR]	*n* (%) or median [IQR]	*n* (%) or median [IQR]	
***Demographics***						
Women	1045 (26.0)	64 (21.8)	285 (24.7)	256 (22.3)	440 (30.8)	**
Age group (years)						**
*18 to 25*	1785 (44.4)	140 (47.8)	587 (50.9)	440 (38.4)	618 (43.3)	
*26 to 45*	1830 (45.5)	118 (40.3)	477 (41.4)	548 (47.8)	687 (48.1)	
> *45*	404 (10.0)	35 (11.9)	89 (7.7)	158 (13.8)	122 (8.5)	
Education level ≥ secondary school diploma	3294 (82.0)	249 (85.0)	932 (80.8)	898 (78.4)	1215 (85.1)	**
Place of residence during lockdown						**
*Rural*	607 (15.1)	44 (15.0)	205 (17.8)	178 (15.5)	180 (12.6)	
*Semi-urban*	1005 (25.0)	81 (27.6)	340 (29.5)	294 (25.6)	290 (20.3)	
*Urban*	2246 (55.9)	149 (50.8)	562 (48.7)	628 (54.8)	907 (63.6)	
No. of people living in the house (incl. participant) during lockdown	2 [2–4]	3 [2–4]	3 [2–4]	2 [2–4]	2 [2–4]	ns
External space at home (terrace, garden) during lockdown	2754 (68.5)	218 (74.4)	816 (70.8)	805 (70.2)	915 (64.1)	**
Living with a partner during lockdown	1716 (42.7)	98 (33.4)	430 (37.3)	545 (47.6)	643 (45.1)	**
Had children during lockdown	956 (23.8)	61 (20.8)	230 (19.9)	345 (30.1)	320 (22.4)	**
Change in employment activity/status during lockdown						**
*Teleworking*	625 (15.5)	36 (12.3)	146 (12.7)	184 (16.1)	259 (18.1)	
*Partial unemployment or receiving payroll subsidy, job loss, sick leave or disability leave*	1667 (41.5)	106 (36.2)	427 (37.0)	468 (40.8)	666 (46.7)	
*Unchanged*	939 (23.4)	78 (26.6)	283 (24.5)	328 (28.6)	250 (17.5)	
*Student*	666 (16.6)	68 (23.2)	252 (21.9)	132 (11.5)	214 (15.0)	
***State of health before lockdown***						
Chronic or acute disease (physical health)	522 (13.0)	39 (13.3)	140 (12.1)	155 (13.5)	188 (13.2)	ns
Chronic or acute disease (mental health)	385 (9.6)	38 (13.0)	124 (10.7)	93 (8.1)	130 (9.1)	ns
Chronic pain	853 (21.2)	57 (19.4)	238 (20.6)	258 (22.5)	300 (21.0)	ns
Self-medication with cannabis						**
*Not always*	2783 (69.2)	198 (67.6)	819 (71.0)	752 (65.6)	1014 (71.1)	
*Always*	436 (10.8)	32 (10.9)	110 (9.5)	164 (14.3)	130 (9.1)	
*Missing*	800 (19.9)	63 (21.5)	224 (19.4)	230 (20.1)	283 (19.8)	
***Substance use***						
Pre-lockdown cannabis use—median number of daily intakes (i.e., frequency)	4 [3–6]	4 [2–5]	4 [3–6]	4 [3–6]	4 [2–5]	ns
Pre-lockdown cannabis form						ns
*Dried flowers*	2518 (62.6)	168 (57.3)	712 (61.7)	737 (64.3)	901 (63.1)	
*Resin*	1460 (36.3)	122 (41.6)	429 (37.2)	393 (34.3)	516 (36.2)	
*Other/missing*	41 (1.0)	3 (1.0)	12 (1.0)	16 (1.4)	9 (0.6)	
Pre-lockdown cannabis route of administration						**
*Smoking*	3845 (95.7)	286 (97.6)	1107 (96.0)	1072 (93.6)	1380 (96.7)	
*Other*	168 (4.2)	7 (2.4)	44 (3.8)	73 (6.4)	44 (3.1)	
Pre-lockdown home-grown cannabis	389 (9.7)	9 (3.1)	71 (6.2)	177 (15.4)	132 (9.2)	**
Pre-lockdown stocking up on cannabis	1859 (46.3)	31 (10.6)	489 (42.4)	525 (45.8)	814 (57.0)	**
Increased tobacco use during lockdown	913 (22.7)	114 (38.9)	311 (27.0)	149 (13.0)	339 (23.8)	**
Increased alcohol use during lockdown	1180 (29.3)	119 (40.6)	323 (28.0)	249 (21.7)	489 (34.3)	**
Increased use of benzodiazepines during lockdown	116 (2.9)	17 (5.8)	35 (3.0)	18 (1.6)	46 (3.2)	**
***Health outcomes during lockdown***						
Symptoms suggestive of COVID-19						**
*No*	2578 (64.1)	205 (70.0)	735 (63.7)	789 (68.8)	849 (59.5)	
*Mild symptoms without dyspnea*	1026 (25.5)	64 (21.9)	285 (24.7)	264 (23.0)	413 (28.9)	
*Dyspnea*	279 (6.9)	16 (5.5)	82 (7.1)	56 (4.9)	125 (8.8)	
Anxiety (HAD score ≥ 8)	1384 (34.4)	111 (37.9)	394 (34.2)	324 (28.3)	555 (38.9)	**
Depression (HAD score ≥ 8)	840 (20.9)	98 (33.4)	169 (14.7)	243 (21.1)	330 (23.1)	**
Sleep disorder intensification	1666 (41.4)	194 (66.2)	535 (46.4)	343 (29.9)	594 (41.6)	**
Increased pain	760 (18.9)	83 (28.3)	222 (19.2)	181 (15.8)	274 (19.2)	**

IQR: interquartile range; ns: non-significant; Missing data and non-responses displayed if > 10%; HAD: Hospital Anxiety Depression

**p < 0.01

### Changes in cannabis use

With regard to changes in cannabis use between before and during the first lockdown, 293 (7.3%) participants stopped, 1153 (28.7%) decreased, 1146 (28.5%) did not change, and 1427 (35.5%) increased their use. The latter reported a median of 2 (IQR: 1–4) additional intakes per day. The characteristics of respondents according to cannabis use changes are presented in Table 1. Several differences were found between the four cannabis user groups hereafter called ‘stopped’, ‘decreased’, ‘unchanged’ and ‘increased’. Among the statistically significant and most marked differences, we found more women in the increased group (30.8% vs. from 21.8 to 24.7%). The largest proportion of respondents aged over 45 years old was in the unchanged group (13.8% vs from 7.7 to 11.9%). Students were more frequent in the stopped group (23.2% vs. from 11.5 to 21.9%) and more people were teleworking (18.1% vs. from 12.3 to 16.1%) and unemployed (46.7% vs. from 36.2 to 40.8%) (whether partial or total) in the increased group. Respondents in the unchanged group were more likely to use cannabis exclusively for self-medication (14.3% vs. from 9.1 to 10.9%), to use a route of administration other than smoking (6.4% vs. from 2.4 to 3.8%), and to use home-grown cannabis (15.4% vs. from 3.1 to 9.2%). No differences were found in pre-lockdown cannabis use frequency (*i.e.,* median number of daily intakes) across the four groups. Similarly, the prevalences of pre-lockdown mental illness, physical illness, and chronic pain were similar across groups. Stocking up on cannabis before the lockdown was widely reported in the increased group (57.0%) but not so in the stopped group (10.6%). Increased tobacco, alcohol and benzodiazepine use was less likely in the unchanged group. Finally, the unchanged group had the lowest rates of anxiety, sleep disorder intensification and increased pain.

### Correlates of changes in cannabis use during lockdown

In the multivariable multinomial model (Table 2), we present adjusted relative-risks ratios (RRR) associated with changes in cannabis use, the ‘unchanged’ group being the reference.

**Table 2:** Association between changes in cannabis use and health outcomes during France’s first COVID-19-related lockdown: multivariable logistic model

	Stopped (*vs.* unchanged)		Reduced (*vs.* unchanged)		Increased (*vs.* unchanged)	
	aRRR [IC 95%]	*p*	aRRR [IC 95%]	*p*	aRRR [IC 95%]	*p*
**Female**	**0.71 [0.50, 1.00]**	**0.050**	0.91 [0.74, 1.12]	0.385	**1.34 [1.10, 1.62]**	**0.003**
**Age group (years)**						
*18 to 25*	0.84 [0.50, 1.41]	0.512	**1.61 [1.16, 2.24]**	**0.005**	**1.45 [1.07, 1.97]**	**0.018**
*26 to 45*	0.88 [0.55, 1.41]	0.593	**1.44 [1.06, 1.95]**	**0.021**	**1.39 [1.05, 1.84]**	**0.021**
> *45*	Ref		Ref		Ref	
**Education level ≥ secondary school diploma**	**1.67 [1.11, 2.51]**	**0.014**	1.19 [0.94, 1.50]	0.155	**1.29 [1.03, 1.63]**	**0.028**
**Having an external space (terrace, garden)**	**1.52 [1.09, 2.11]**	**0.013**	1.13 [0.93, 1.37]	0.206	0.88 [0.74, 1.06]	0.178
**Type of area of residence (during lockdown)**						
*Rural*	Ref		Ref		Ref	
*Semi urban*	1.18 [0.75, 1.85]	0.482	0.97 [0.74, 1.27]	0.813	0.94 [0.72, 1.24]	0.686
*Urban*	1.00 [0.66, 1.53]	0.989	**0.73 [0.57, 0.94]**	**0.016**	1.22 [0.95, 1.57]	0.112
**Living with a partner during lockdown**	**0.72 [0.52, 0.98]**	**0.036**	**0.81 [0.68, 0.98]**	**0.026**	0.96 [0.80, 1.13]	0.605
**Employment during lockdown**						
*Teleworking*	0.91 [0.56, 1.49]	0.718	0.95 [0.71, 1.26]	0.716	**1.52 [1.16, 1.98]**	**0.002**
*Partial unemployment or receiving payroll subsidy, job loss, sick leave or disability leave*	0.98 [0.69, 1.41]	0.929	1.01 [0.81, 1.25]	0.944	**1.76 [1.43, 2.18]**	**< 0.001**
*Unchanged*	Ref		Ref		Ref	
*Student*	**2.19 [1.36, 3.52]**	**0.001**	**1.77 [1.31, 2.39]**	**< 0.001**	**1.69 [1.24, 2.29]**	**0.001**
**Used cannabis exclusively to self-medicate**						
*No*	Ref		Ref		Ref	
*Yes*	0.77 [0.47, 1.25]	0.289	**0.62 [0.46, 0.83]**	**0.002**	**0.64 [0.48, 0.85]**	**0.002**
**Home-grown cannabis**	**0.19 [0.09, 0.39]**	**< 0.001**	**0.46 [0.34, 0.63]**	**< 0.001**	0.90 [0.69, 1.18]	0.456
**Pre-lockdown cannabis stockpiling**	**0.11 [0.08, 0.17]**	**< 0.001**	**0.78 [0.65, 0.93]**	**0.006**	**1.38 [1.17, 1.64]**	**< 0.001**
**Increased tobacco use (during lockdown)**	**3.07 [2.22, 4.24]**	**< 0.001**	**2.21 [1.76, 2.77]**	**< 0.001**	**1.79 [1.44, 2.24]**	**< 0.001**
**Increased alcohol use (during lockdown)**	**2.00 [1.47, 2.71]**	**< 0.001**	**1.29 [1.05, 1.58]**	**0.014**	**1.61 [1.34, 1.95]**	**< 0.001**
**Increased benzodiazepine use (during lockdown)**	**2.32 [1.08, 4.99]**	**0.031**	1.57 [0.86, 2.89]	0.144	1.67 [0.93, 2.99]	0.085
**Symptoms suggestive of COVID-19**						
*No*	Ref		Ref		Ref	
*Mild symptoms without dyspnea*	0.75 [0.53, 1.06]	0.106	1.04 [0.85, 1.28]	0.683	**1.28 [1.05, 1.55]**	**0.013**
*Dyspnea*	1.26 [0.67, 2.37]	0.471	**1.51 [1.04, 2.21]**	**0.031**	**1.88 [1.32, 2.68]**	**< 0.001**
**Self-reported pre-lockdown chronic pathology or anxiety/depression**						
*No pathology*	Ref		Ref		Ref	
*Pathology other than anxiety or depression*	0.80 [0.46, 1.39]	0.434	1.10 [0.80, 1.49]	0.558	1.2 [0.90, 1.60]	0.213
*Anxiety or depression*	**1.75 [1.02, 3.01]**	**0.043**	**1.53 [1.05, 2.23]**	**0.025**	1.02 [0.70, 1.47]	0.931
**Self-reported pre-lockdown chronic pain**	**0.51 [0.33, 0.77]**	**0.002**	0.84 [0.66, 1.08]	0.174	0.89 [0.71, 1.13]	0.335
**Depression during lockdown (HAD score > = 8)**	**1.46 [1.02, 2.07]**	**0.036**	1.06 [0.83, 1.35]	0.660	**1.34 [1.07, 1.69]**	**0.011**
**Increased pain during lockdown**	**1.90 [1.31, 2.75]**	**0.001**	1.11 [0.86, 1.42]	0.424	1.00 [0.79, 1.27]	0.988
**Sleep disorder intensification during lockdown**	**3.51 [2.56, 4.82]**	**< 0.001**	**1.80 [1.49, 2.18]**	**< 0.001**	**1.35 [1.13, 1.62]**	**0.001**

aRRR: adjusted relative-risk ratio; 95%CI: 95% confidence interval; p: p-value, HAD: Hospital Anxiety Depression, in bold : *p* < 0.05

Female gender and younger age were correlated with increased cannabis use as was having at least a secondary school diploma. Being a student, having switched to teleworking, and unemployment were all positively associated with increased cannabis use compared with persons with unchanged working practices. With regard to cannabis use, those who increased their number of intakes were less likely to use the drug exclusively for self-medication and were more likely to have stocked-up on it in expectation of the lockdown. They were also more likely to have increased tobacco, alcohol and benzodiazepine use during the lockdown. Finally, they were more likely to have experienced COVID-19-related symptoms, depression and sleep disorders during the lockdown.

Compared with participants with an unchanged level of cannabis use, those who decreased their use without stopping it were more likely to be under 45 and to be students. Instead, they were less likely to live in urban areas and to live with a partner. They were also less likely to use cannabis to self-medication, to grow cannabis at home and to have made pre-lockdown cannabis stock. Increased tobacco and alcohol use during lockdown was associated with decreased cannabis use. They were also more likely to self-report pre-lockdown anxiety or depression and to have experienced dyspnea, and sleep disorders intensification during lockdown.

Respondents who stopped cannabis use were less likely to be women and to be living with a partner, but more likely to have at least a secondary school diploma, to be a student and to have an external space in their house. They were less likely to grow cannabis at home and to have stocked-up cannabis before the first lockdown, but more likely to have increased tobacco, alcohol and benzodiazepine use during the lockdown. Furthermore, they were more likely to self-report pre-lockdown anxiety or depression, but less likely to report pre-lockdown chronic pain. Finally, they were more likely to have experienced depression, pain increase and sleep disorders intensification during lockdown.

## Discussion

To our knowledge, this is the first study to investigate the impact of COVID-19 lockdown measures on a large sample of daily CU in France. In a context where all cannabis use was prohibited in the country at the time of the study, our findings reveal that the majority of respondents changed their cannabis use patterns. Specifically, over a third increased their daily number of intakes, while 36% decreased or completely stopped their use. These results are in line with a previous study performed in France from day 8 to day 13 (i.e., 24 March to 29 March 2020) of the first lockdown among 620 CU (frequency of intakes unknown) with 39.5%, 31.2% and 29.3% reporting no change, an increase, and decrease/cessation, respectively [4]. In the Netherlands, where cannabis-vending coffee shops were open for takeaway purchases during that country’s first lockdown a transversal study was performed among 1563 CU, 67.9% of whom were daily or almost daily users. That study showed that 41.3% of respondents reported increased cannabis use, 49.4% no change, and 6.6% a decrease [30]. Similarly, 38.4% of a sample of 1202 medical CU in the United States reported an increase in cannabis use since the start of the pandemic, 47.9% no change, and only 8.8% a decrease [25]. This suggests that the availability of cannabis is an important predictor of change in patterns of use. In our model, using home-grown cannabis and having stocked-up cannabis were both negatively associated with a decrease or cessation of cannabis use. Furthermore, almost everyone in our sample who reported stopping cannabis use during France’s first lockdown did not stock up on the drug beforehand. Their cessation might therefore be explained by supply shortage. Conversely, those who increased their cannabis use were more likely to have stocked up. Interestingly, respondents in the ‘unchanged’ category were more likely to report home-grown cannabis for self-use, which suggests that self-supply may help when faced with difficulties in accessing cannabis. It must be remembered however that in France, growing one’s own supply means risking legal ramifications. Other countries have recently decided to legalize its recreational use. One example is Canada, where the effects of this policy are currently being evaluated [31].

We found other correlates of changes in cannabis use. Female gender and younger age were significantly associated with increased cannabis use, which is consistent with previous studies [4, 30]. In France, universities were closed during the first lockdown, and we found that being a student was significantly associated with an increase in cannabis use, but also a decrease and even cessation, indicating the differential impact of the lockdown on students. Working practices were also abruptly impacted by the lockdown, specifically an increase in teleworking and in unemployment [32]. In our study, persons who became unemployed during the lockdown were more likely to report an increase in cannabis use than those whose working practices remained unchanged. Using cannabis exclusively to self-medicate was a protective factor against increased or decreased cannabis use, suggesting that such CU have more stable consumption patterns and were less impacted by the lockdown and supply problems.

With regard to health outcomes in our study sample during the first lockdown, unchanged cannabis use was associated with lower prevalences of depression, sleep disorder intensification, and increased pain. One hypothesis for this is that for those who increased their cannabis use, this increase was a response to adverse health outcomes participants experienced, as cannabis is commonly used to therapeutically cope with these issues [33, 34]. Conversely, stopping cannabis use was associated with a higher prevalence of these three health outcomes, even after adjustment for various potential confounders. This is not surprising as these symptoms are typical of cannabis withdrawal [35]. This association suggests that cannabis cessation was more forced than desired for a proportion of the respondents. In addition, increased use of other psychoactive substances—specifically tobacco, alcohol and benzodiazepines—was more frequent in the group which stopped cannabis use. More research is needed to evaluate whether these changes in consumption continued after the first lockdown ended. In the general population, mental health was significantly affected by the pandemic, especially during lockdown periods [36]. Thus, links between mental health outcomes and cannabis use changes might have been influenced by the crisis itself. This may explain the meaningful increase of alcohol use across all groups of cannabis changes. Overall, our results reflect a complex interplay between an unprecedented health crisis, drastic changes in life habits and cannabis supply shortage on cannabis use. It is difficult to discern whether changes in cannabis use were forced or deliberate. As designed, our cross-sectional study does not address causality and repeated studies would help to understand these relationships.

In the context of the ongoing COVID-19 pandemic, lessons can be drawn from our study as well as several public health and social implications. First, implementation of mental health care and counselling towards people with cannabis use disorder could have been beneficial to mitigate deleterious outcomes. Moreover, clinicians should now be aware of the impact of COVID-19-related lockdowns and be encouraged to tailor their responses to people who present mental and/or physical health problems associated with changes in cannabis use. Moreover, given that other studies have reported difficulties for patients to physically attend healthcare facilities during COVID-19 lockdowns, and the familiarity of CU with digital technologies, the development of telehealth could be advocated [37, 38]. Socio-environmental changes are also needed as a great deal of stigma still surrounds cannabis use and users in France, and this represents a barrier to healthcare [39]. Second, we may question whether making cannabis available as an “essential good” could have prevented abrupt cessation of cannabis use and further impact on health and other drug use, including alcohol and benzodiazepines.

Our study has limitations. First, the online recruitment method used may have led to selection bias, in particular underrepresentation of people not proficient in computing skills and those without internet access. The representativeness of our sample can only be partially assessed by comparing our results with those from the only other French study to include daily CU to date [12]. Although that study—based on a representative sample of the general population—only detailed gender and age distribution, the values for socio-demographic characteristics found were similar to ours (*i.e.,* mostly men and a younger population). Second, to ensure the length of the questionnaire was acceptable, we chose only a small number validated scales and used only one question to measure pain increase and one for intensification of sleep disorders. Third, given that cannabis use is illegal in France, individuals may have been reluctant to participate due to fear of legal actions. However, we guaranteed complete anonymity through our secured platform. Finally, the cross-sectional nature of our data prevented us from making assumptions about causal relationships. Nevertheless, our findings provide useful information about the impact of the first COVID-19 lockdown in France on daily CU.

## Conclusions

We highlighted the differential impact of France’s first COVID-19-related lockdown on daily CU. Cannabis use patterns changed for the majority of respondents, with 35.5% increasing and 36% decreasing or stopping their use. We found several factors associated with changes in cannabis use, providing us with a greater understanding of the behavioral and health consequences of COVID-19-lockdowns in daily CU. In a context where the pandemic is still ongoing, these findings could be very useful for clinicians and decision-makers when designing and implementing strategies to mitigate adverse health outcomes, including expanded access to healthcare, harm reduction interventions and policy changes.

## Data Availability

The datasets used and analyzed for the current study are available from the corresponding author on reasonable request (perrine.roux@inserm.fr).

## Abbreviations

COVID-19: Coronavirus disease 2019
CU: Cannabis users
IRB: Institutional Review Board
AUDIT-C: Alcohol Use Disorders Identification Test
INSEE: French National Institute of Statistics and Economic Studies
HAD: Hospital and Anxiety Hospital
IQR: Interquartile range
RRR: Relative-risks ratios
IORG: International Organizations Research Group
FWA: Federal wide Assurance
